# Robotic-assisted lung nodule diagnosis and resection

**DOI:** 10.3389/fonc.2025.1555151

**Published:** 2025-03-21

**Authors:** Priya P. Patel, Ami Patel, Benjamin Zollinger, Kei Suzuki

**Affiliations:** ^1^ Department of Surgery, Division of Thoracic Surgery, Schar Cancer Institute, Inova Health System, Fairfax, VA, United States; ^2^ Department of Pathology and Laboratory Medicine, New York Presbyterian Hospital/Weill Cornell Medicine, New York, NY, United States

**Keywords:** bronchoscopy, lung cancer, lobectomy, lung nodule, resection, robotic

## Abstract

In the care of lung cancer patients, early diagnosis followed by timely therapeutic procedures can have a significant impact on overall survival and patient anxiety. While robotic-assisted lung resection is now a widely accepted surgical approach, robotic-assisted bronchoscopy is a more recent diagnostic procedure that improves reach, stability, and precision in the field of bronchoscopic lung nodule biopsy. The ability to combine lung cancer diagnostics with curative-intent surgical resection into a single-setting anesthesia procedure has the potential to decrease costs, improve patient experiences, and most importantly, reduce delays in cancer care. In addition, with the expected adoption of sublobar resection for stage I lung cancer ≤2cm, combining robotic-assisted bronchoscopy with robotic surgery offers a single-setting pathway to take advantage of the precision biopsy and localization technique offered by robotic-assisted bronchoscopy and the precision operation offered by robotic surgery. We herein describe our approach to this single-setting procedure. While limited studies suggest that the combined approach results in shorter overall operative time and cost, we need future work to better characterize the overall operative time, complication rates, long-term oncologic outcomes, and cost analysis.

## Introduction

With advances in technology and initiation of lung cancer screening programs, the frequency of localized lung cancer amenable to curative intent has increased. Though only accounting for a small portion of total lung cancer diagnoses, the 5-year survival for only localized lung cancer is 65% (1). A vital part of lung cancer management is minimizing the time from diagnosis to therapeutic treatment. Delays in treatment, which are defined as resection 8 weeks or more after diagnosis, are associated with higher rates of upstaging, increased 30-day mortality, and decreased median survival (2). Delays in treatment are also accompanied with higher overall healthcare resource utilization and cost. Initiation of diagnostic workup does not always immediately yield a tissue diagnosis either. 46% of lung cancer patients will require two or more biopsies. In such cases, wedge resection for surgical biopsy can be required. Despite being a more invasive option, these can be in turn associated with 10-20% benign resection rate (3). Therefore, the ability to combine precise diagnostic procedures and therapeutic surgical resection, hence reducing delays and resultant rates of upstaging at diagnosis, may allow for less resource utilization and eventual cost. Higher stages have been shown by retrospective analysis to be associated with significantly higher total healthcare costs (4).

In the management of lung nodule/cancer, robot-assisted bronchoscopy (RAB) is an emerging technology to biopsy lung lesions. Combining RAB with robot-assisted thoracoscopic surgery (RATS) offers an approach that takes advantage of the two technologies (5). With the changing landscape of early-stage lung cancer treatment, combining RAB with RATS may offer several advantages with the primary benefit being single setting lung cancer diagnostics, mediastinal staging and definitive treatment while expediting overall lung cancer care. We herein review our approach to single setting RAB/RATS and various factors in this combined setting, such as cost and outcomes.

## Lung cancer diagnostics

**Figure 1:**
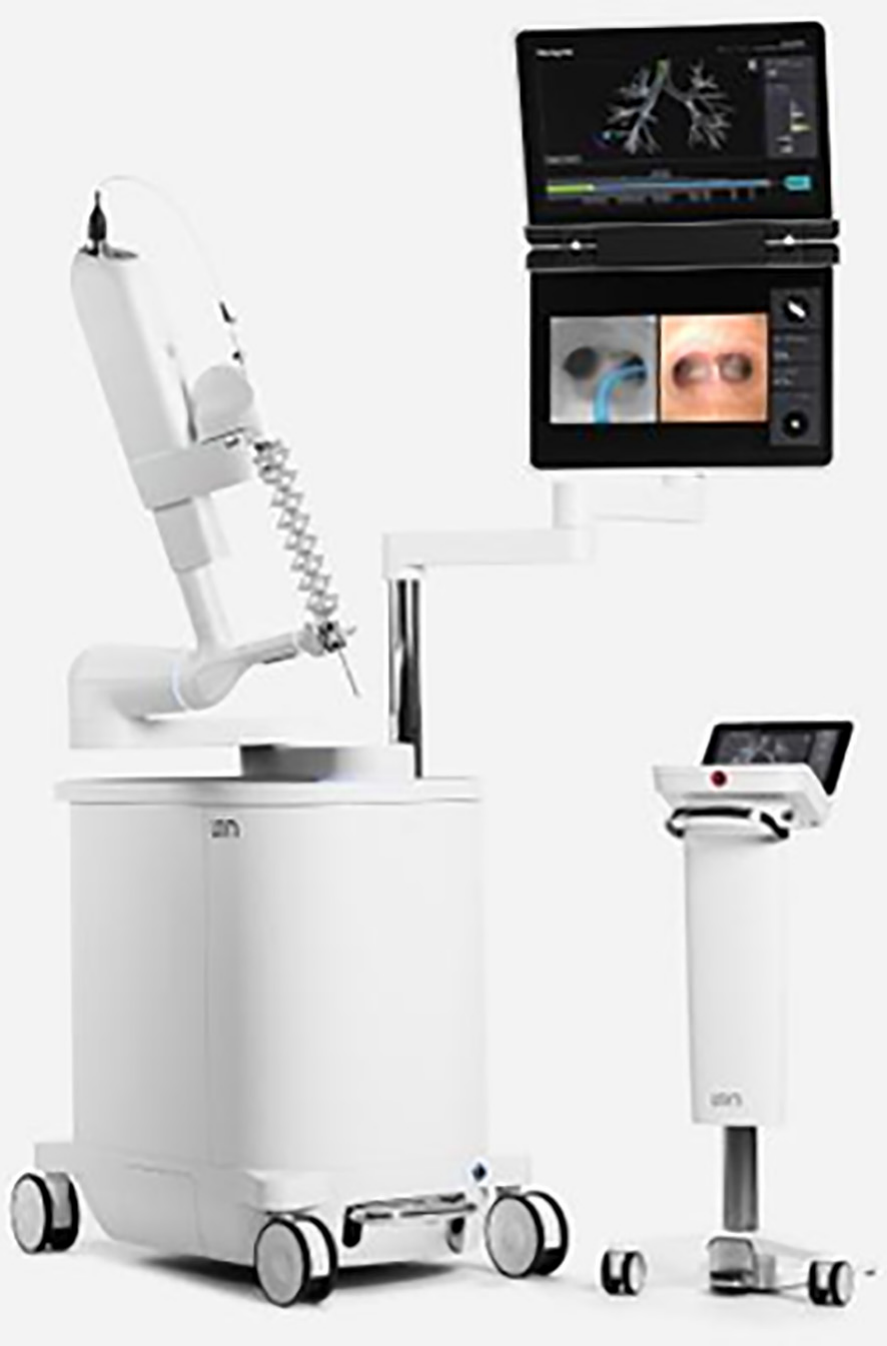
Ion robotic-assisted endoluminal platform (Intuitive Surgical, Inc).

**Figure 2:**
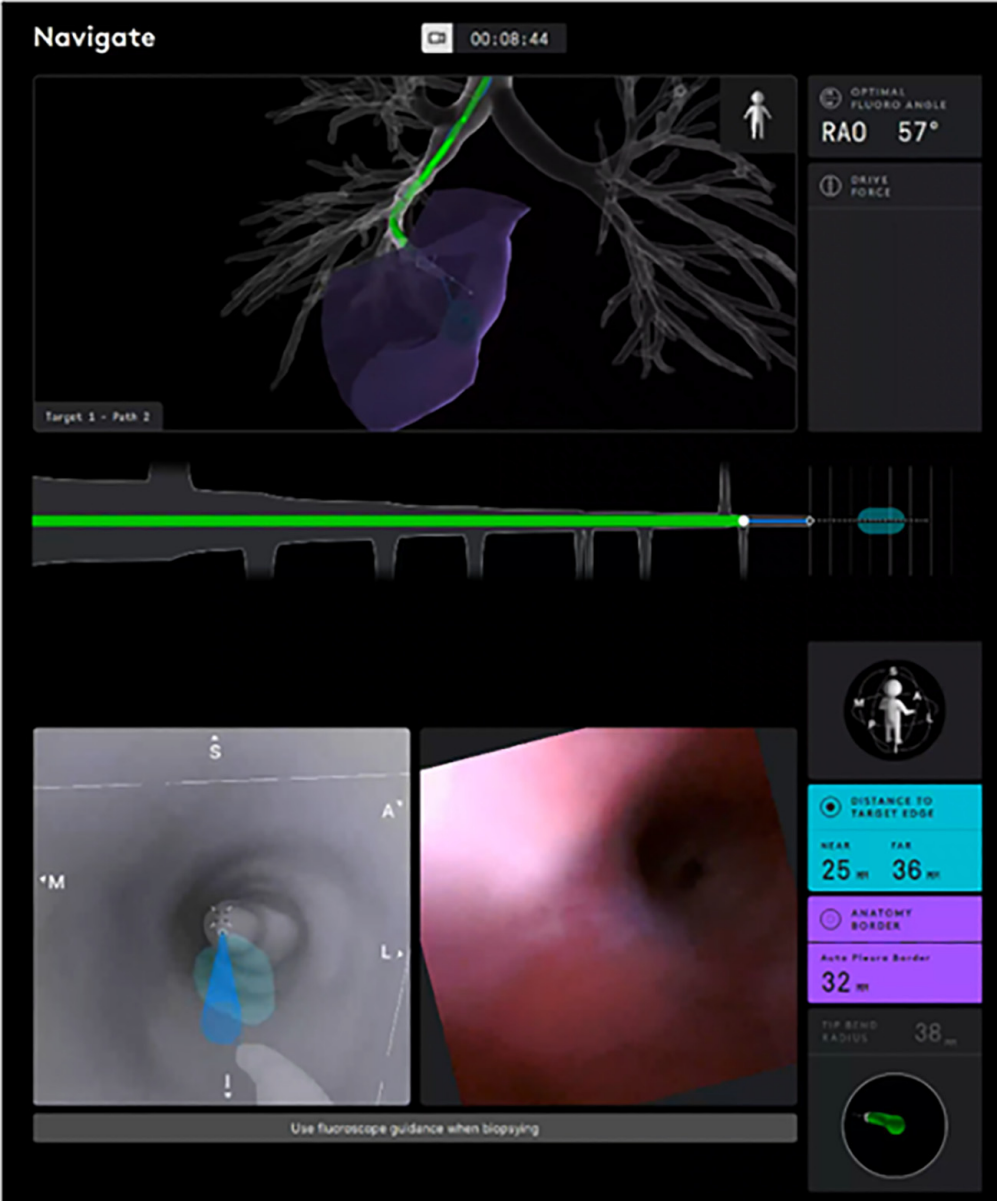
Robotic assisted bronchoscopy is designed to allow endobronchial navigation into the lung periphery while allowing direct visualization of peripheral airways and maintaining catheter stability and shape to maximize precision during sampling.

The American College of Chest Physicians and National Comprehensive Cancer Network guidelines both recommend obtaining a diagnosis of the primary lesion, resultant staging, and tissue for molecular testing by using the least invasive modality. Ideally, this would be a single procedure (6, 7). An initial bronchoscopic approach allows for biopsies of both the primary lesion of interest as well as mediastinal nodes for staging. The set of bronchoscopic procedural tools and technologies that aid in airway navigation, confirmation of target proximity, and tissue sampling are collectively known as guided bronchoscopy (8). The main components of guided bronchoscopy comprise image-mapped airway navigation, videoscopic real-time airway visualization, intraprocedural confirmatory imaging, and specimen acquisition devices. Studies have defined various metrics of guided bronchoscopy (9–16), defining diagnostic yield and safety, and comparing guided bronchoscopy methods with percutaneous sampling, consistently finding it to be safer relative to transthoracic sampling (17, 18). One published downside of the bronchoscopic approach is the diagnostic yield, which has been inconsistent in randomized controlled trials, with yields from 44% to 74%, compared with rates above 90% for percutaneous sampling (10–18). The novel technology of RAB allows us to overcome this main limitation, and there are different available platforms (19, 20). The Ion Robotic-Assisted Endoluminal Platform (Intuitive Surgical, Inc.) is based on novel shape-sensing RAB (ssRAB) technology (**Figure 1**) (21, 22). The Monarch RAB platform (Auris Health, Inc.) is based on electromagnetic navigation (EMN RAB) technology (23). RAB is designed to allow navigation into the lung periphery via endobronchial approach. The robotic control allows for increased catheter stability and guidance to maximize accuracy and precision for biopsy while still providing direct and/or virtual visualization of peripheral airways with simultaneous computer mapping of the catheter’s path and target (**Figure 2**). These advantages of RAB have been demonstrated in cadaveric models and subsequently supported by several clinical studies demonstrating a navigational success rate between 96.2% to 100% (24–31). Diagnostic yield for malignancy has been shown to be 69.1% using EMN RAB and as high as 88% using ssRAB, improved from comparative non-robotic bronchoscopic approaches for peripheral lesions (24–29, 31). The RAB technology is also well positioned to allow for integration with other existing valuable resources for the diagnosis, staging, and treatment of lung lesions such as endobronchial ultrasound (EBUS), radial EBUS, fluoroscopy, cone beam computed tomography and the da Vinci Surgical System.

Another advantage of RAB is its ability to definitively mark small peripheral lung lesions for eventual resection. Especially with improved efforts in lung cancer screening, we will encounter more small peripheral lesions, often ground glass in nature. Identifying these lesions intra-operatively can frequently pose a challenge, and the ability to localize these lesions pre-operatively can be useful, especially when this can be done at the same time as the surgical resection. Current methods for marking lung nodules include coils, hook-wire, or radiotracer placement percutaneously placed under CT guidance by interventional radiologists. Although these transthoracic methods have been found to be effective in delivering their markers, the higher rate of pneumothorax, bleeding, hematoma and dislodgement of tracer are noted limitations when compared to bronchoscopic approach (32, 33). Furthermore, these techniques and interventions require coordination and scheduling across multiple specialists and frequently result in significant delay to surgical resection (32–36). Bronchoscopic lung nodule marking has been found to be effective and safe in comparison with other methods (37, 38). Available techniques for peripheral lung nodule marking using RAB include but are not limited to dye marking (using methylene blue, indocyanine green or iopamidol) or fiducial marker placement.

## On-site diagnosis

Based on the systematic review and expert panel convened by the College of American Pathologists (CAP), rapid on-site evaluation (ROSE) is recommended, if available in EBUS- transbronchial needle aspiration (TBNA) and strongly recommended if clinically feasible in transthoracic needle procedures (39). Oki et al. performed a prospective randomized clinical trial on patients undergoing EBUS- TBNA and showed a statistically significant reduction in the need for additional procedures with ROSE (11% of patients in ROSE vs 57% in non-ROSE group; p<0.001) (40). ROSE has also shown to slightly improve diagnostic yield (absolute percentage increase of 2.9% – 8%) (41–43). In addition, studies *suggest* ROSE can ensure the adequacy of material obtained for ancillary studies and minimize molecular testing failures (44, 45). However, the lack of trained personnel, time commitment and other resources associated with ROSE may act as barriers against its use and thus may not be available in all hospital settings.

## Lung cancer resection

Another instance in which RAB/RATS combination may be beneficial is in the treatment of ≤2cm lesions, which we expect to see more of with improved lung cancer screening. With the recent findings from the JCOG 0802 and CALGB 140503 studies, sublobar resection for stage I lung cancer ≤2cm has become an acceptable surgery (46, 47). Hence, we may see an increase in the number of sublobar cases being performed for ≤2cm lesions, further emphasizing the importance of precision biopsy as well as the need for reliable localization technique to aid with the surgery. Segmentectomy can be a more technically demanding operation compared to lobectomy as it requires familiarity of the segmental anatomy. Features of the robotic platform may mitigate the technical demands of more distal dissection that is required in performing segmentectomy. In conjunction with the improved diagnostic tool in RAB, we may be able to obtain accurate diagnosis even in these small lesions, which subsequently could increase the number of segmentectomies being performed.

## Single setting anesthesia event

### Pre-operative evaluation

Preoperative evaluation is performed in a standard fashion, including CT and positron emission tomography (PET). We prefer a thin slice (1.0mm slices) chest CT imaging for pre-procedural planning and staging purposes. Pathway to the nodule is planned based on thin slice chest CT and can be done before the actual procedure. The patient is consented for both the RAB and RATS (in case if malignancy is confirmed on examination intraoperatively). Both procedures will be completed under a single anesthesia event (5).

### Pre-operative selection

Patient selection for the single anesthetic pathway is essential, and one must consider optimizing operating room block time, minimizing occult N2 nodal disease rate, and limiting benign resections. Although no published criteria exist for the single anesthetic pathway, the following are criteria our program follows:

- Nodule suspicious for NSCLC and ≤3cm.
- No chest wall or surrounding structure invasion which would make the lesion ≥T3.
- Clinical stage 1-2 NSCLC.
- No mediastinal adenopathy by CT nor PET criteria.
- Adequate PFTs with good performance status for the proposed resection.
- Patients who travel from far distance to receive treatment.
- Agreeable and trusting patient.

### Team training and preparation

As the single anesthesia RAB/RATS approach requires multi-disciplinary approach, pre-procedure planning is of paramount importance. We have a workflow in which CT scans are performed per robotic navigational bronchoscopy protocols with the appropriate slice thickness and amount of overlap. Essential to this approach is a pathology team, as the cytologist not only has to make intraoperative judgements on tissue adequacy but also provide a diagnosis of malignancy or benign. Discussion with the anesthesia team involves single lumen intubation for RAB portion and double lumen for RATS. Communication between the interventional pulmonologist and thoracic surgeon can have various permutations. The pulmonologist may first perform the RAB biopsy and EBUS, then the surgeon performs the RATS in the same room if the set-up allows. Some have done RAB biopsy and EBUS in a bronchoscopy or endoscopy suite and then move the patient to the operating room for resection, while most will perform all stages of the single anesthetic event in the operating room.

### Intraoperative methods

**Figure 3:**
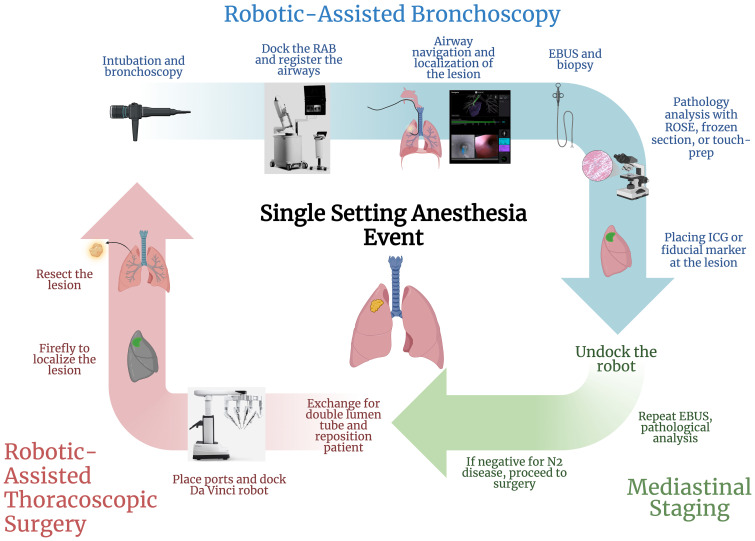
Protocolized flowchart of a single-setting robotic-assisted bronchoscopy and thoracic surgery (created with BioRender.com). RAB, robot-assisted bronchoscopy; EBUS, endobronchial ultrasound; ROSE, rapid on-site evaluation; ICG, indocyanine green.

We describe our intraoperative RAB/RATS workflow below (**Figure 3**).

### Part I: RAB

Patient is placed under general anesthesia with muscle paralysis in the supine position. Patient is then intubated with a single lumen endotracheal tube (ETT), at least 8.0 in size. We perform a standard flexible bronchoscopy to ensure the tip of ETT is at least 2-3 cm from the carina and to clear any secretions.

The robot is docked to an endotracheal tube adapter. The virtual bronchoscopy is registered to the patient’s airway in real time by marking the main carina as well as each subsegment of the upper and lower lobes. After registration, navigation begins using real-time bronchoscopic view of the patient’s airways complemented with a virtual bronchoscopy image, which navigates the user towards the lesion. When the target lesion is within 1-3cm from the tip of the catheter, the vision probe is removed (when using Ion Endoluminal System), and a radial endobronchial ultrasound (R-EBUS) is utilized to identify and confirm lesion. If biopsy requires traversing a cartilaginous bronchial wall, a needle is first used to puncture the wall to create a path for the R-EBUS probe. Once confirmed, biopsies are taken under image guidance with tools such flexible needles for fine needle aspiration (FNA) samples and forceps for frozen section specimens. Adjunct imaging to help with the biopsy include 2D fluoroscopy, 3D fluoroscopy, or cone-beam CT scan. Cytology slides from FNA samples can be read with ROSE. Forcep specimens can be processed by frozen section for intraoperative interpretation or by touch-prep and cytologic interpretation.

Once a diagnosis of malignancy is confirmed, the proceduralist has the option to mark the nodule. Our program uses a mixture of 0.5mL of methylene blue and 0.5mL of ICG. The 1.0mL total volume includes priming of the needle, and it is not necessary to clear the needle with air. This prevents spraying of the dye and maximizes the concentration of the dye at the site of the lesion. Utilization of dye marking may help the surgeon when performing segmentectomies to confirm appropriate anatomic location as well as ensuring adequate margin (48). Thereafter, the optical probe is then inserted again through the catheter to confirm adequate hemostasis. The robotic bronchoscope is then withdrawn and undocked from the ETT.

### Mediastinal staging

Mediastinal staging, if applicable, is then performed, usually with EBUS. ROSE or intraoperative cytology interpretation then confirms the absence or presence of metastatic malignant disease. If the nodule biopsy confirms malignancy and mediastinal staging examination is negative for N2 metastatic disease, the patient in the same setting undergoes RATS for surgical treatment of their lung cancer.

### Part II: RATS

Once the RAB portion is done, the single lumen ETT is exchanged for a double lumen ETT, and the patient is positioned in the lateral decubitus position in preparation for the surgical resection with the da Vinci Surgical. The operative lung is isolated, and the robotic ports are placed. Intraoperatively, the firefly function can be used to locate the lesion, which has been marked.

## Advantages of a single anesthesia event

The combination of both the diagnostic biopsy and resection into a single event offers multiple benefits for patients and surgeons. The ability to place dye to mark the lesion can certainly make visualization of the lesion easier in the subsequent resection. This is particularly important with RATS where the surgeon does not have the tactile feedback of lesions that would be available in a traditional video-assisted thoracoscopic surgery (VATS) or thoracotomy that can help with target lesion localization and instead has to rely solely on visual cues. Furthermore, for ground-glass predominant lesions, tumor palpation can still be difficult, and preoperative localization may help. The dye or fiducial marker from the preceding bronchoscopy can help the surgeon ensure the specimen contains both the target lesion and adequate margins. Similarly, the lesion marking can aid with intra-operative decision making regarding the extent of the resection.

Beyond the technical advantages of the single-setting procedures, the decrease in time interval between biopsy proven diagnosis and resection also has tangible benefits for patients. Increases in time between initial biopsy and subsequent resection have been associated with higher rates of pathologic upstaging and decreased cancer-specific and overall survival (2, 49, 50). Obtaining tissue diagnosis and subsequent definitive resection immediately eliminates any delay in treatment. For patients who live remotely with extensive travel to referral centers with thoracic surgeons or who have other barriers to accessing care, a single episode of care with both procedures certainly can ease the burden on these patients. A single anesthesia event can also be safer for patients with other comorbidities that carry risk with repeated anesthetic induction. Eliminating the time between biopsy and surgery also lessens the natural anxiety associated with carrying a new cancer diagnosis and an impending surgery.

## Clinical outcomes

As the combined RAB/RATS in one setting is a new approach, there is paucity of literature on various clinical and financial outcomes. Study by Palleiko et al. compared their experience of 36 combined RAB/RATS cohort to 35 who underwent standard workup and provides insights into some of the important clinical as well as financial outcomes (51). In their experience, the combined approach added 73 minutes to lobectomies although when looking at total operating room time, the combined approach was 54 minutes shorter (459 minutes for combined vs 513 minutes for standard). Median hospital stay was similar (3 days) as well as postoperative complication rates between the two groups. In addition, the combined approach resulted in lower direct and indirect costs compared to the traditional workup when comparing the lobectomy cohort. Wong et al. reported their series of 15 combined RAB/RATS approach. While they do not compare their cohort to any control, their study provides insight into time in the OR – median of 284 minutes for RAB plus RATS and 355 minutes when EBUS was added (52). For long term oncologic outcomes, we will have to await for future studies to determine if the combined approach is different from standard.

## Conclusion

Lung cancer patients depend on timely diagnosis and treatment. Beyond poorer oncologic outcomes, heavy emotional tolls, increased utilization of health care resources, and increased overall cost of care to patients and the system are all deleterious outcomes from delays in diagnosis and treatment (53). Timeliness is a vital part of delivering quality oncologic care, and it has been named as one of the six dimensions of health care quality by The Institute of Medicine’s Committee on Quality Health Care in America (54). Reflecting this for the treatment of lung cancer specifically, the RAND Corporation (following guidelines by the British Thoracic Society) targeted timeliness for the care of lung cancer (55). These recommendations advocate that lung cancer diagnoses should be established within 2 months after initial abnormal imaging and treatment offered within 6 weeks after diagnosis (55). Subsequently, updated guidelines were proposed in the United Kingdom’s National Optimal Lung Cancer Pathway which set maximum waiting times of 14 days for diagnosis and 28 days for treatment (56). Jacobsen et al. published a comprehensive systematic review on timeliness of lung cancer care and reported a median wait time from PCP referral to specialist consultation of 1 to 17 days, range median delay from diagnosis to treatment of 6 to 45 days, and estimated 15-63% of patients not receiving treatment within 31 days of diagnosis (**Figure 3**) (57). Authors cite within the constituent studies various barriers to care such as numerous procedures, necessary repetitive procedures, poor appointment availability for specialist visits or procedures, and delays due to physicians not initially suspecting cancer (57). These may reflect broader issues across the field with fragmentation of care, sub-specialization, and poor coordination between providers.

Multidisciplinary evaluation and subsequent treatment offer a potential solution that combines both to timely diagnosis and optimal treatment (57, 58). This multidisciplinary approach is reflected by single setting anesthesia events which offer robotic lung nodule diagnosis with immediate RATS. Similarly to how multidisciplinary clinics can eliminate gaps between evaluations by various specialists, a single setting event can eliminate gaps between tissue diagnosis and necessary resection. The use of robotic assisted procedures has increased dramatically over the last decade despite associated costs. Data are continually emerging that robotic assisted procedures may offer improved lung cancer diagnostic capability and surgical outcomes relative to traditional guided bronchoscopy and VATS (24, 29, 30, 59–63). Close coordination of these technologies offers an integration of the diagnosis, staging, and resection of lung cancer. Multiple ongoing studies support the decreasing time from diagnosis to treatment (64, 65). The following are proposed advantages for the single anesthetic pathway to lung cancer care:

- Shortened timeline from nodule identification to surgery.
- Minimizing anxiety interval between diagnosis and resection.
- Potential reduction in risks associated with multiple anesthetic events.
- Enhanced target lesion identification and visualization in RATS to help with sublobar resections.
- Decreased impact on family resources.
- Earlier return to usual lifestyle.
- Possible decreased cost in lung cancer care without compromising oncologic standards.

Clinicians should target diagnostic and treatment modalities for that maximize accuracy and timeliness while minimizing cost and risk. A multidisciplinary approach in diagnosing and treating malignancy can help provide this optimal pathway. Single setting event robotic-assisted lung nodule diagnosis and resection offers a valuable new tool and strategy in the pursuit of improving the multidisciplinary treatment of lung cancer.
